# Long non‐coding RNA VPS9D1-AS1 facilitates cell proliferation, migration and stemness in hepatocellular carcinoma

**DOI:** 10.1186/s12935-020-01741-7

**Published:** 2021-02-24

**Authors:** Xinxin Fa, Ping Song, Yu Fu, Yu Deng, Kai Liu

**Affiliations:** 1 Department of Gastroenterology, Rizhao People’s Hosptial, Rizhao, 276800 Shandong China; 2 Department of Infectious Diseases, Rizhao People’s Hosptial, Rizhao, 276800 Shandong China; 3 Department of Hepatopancreatobiliary Surgery, The First Hospital of Jilin University, 71 Xinmin Street, Changchun, Jilin China

**Keywords:** SEC61A1, Hepatocellular carcinoma, VPS9D1-AS1, miR-491-5p

## Abstract

**Background:**

Hepatocellular carcinoma (HCC) is a common cancer leading to high morbidity and mortality in worldwide. Previous studies revealed that SEC61 translocon alpha 1 subunit1 (SEC61A) can act as an oncogene in colon adenocarcinoma. However, the functions and molecular mechanism associated with HCC progression remain to be explored. This study aimed at exploring the role of SEC61A1 in HCC progression.

**Methods:**

EdU assay and colony formation assay were applied to assess cell proliferation. The migratory ability of transfected HCC cells was evaluated by transwell migration assay. Sphere formation assay was used to detect the stemneess of HCC cells. Bioinformatics analysis tools and mechanism experiments were used to predict and analyze the potential molecular mechanism associated with the upregulation of SEC61A1 in HCC cells.

**Results:**

Up-regulated SEC61A1 facilitated cell proliferation, migration and stemness in HCC cells. MiR-491-5p negatively regulated SEC61A1 and inhibited HCC cell proliferation and migration by targeting SEC61A1. VPS9D1 antisense RNA 1 (VPS9D1-AS1) could up-regulate SEC61A1 through sponging miR-491-5p. Early growth response 1 (EGR1) was identified as the upstream transcriptional activator for both SEC61A1 and VPS9D1-AS1.

**Conclusions:**

Our study unveiled a novel molecular pathway facilitating HCC cell proliferation, migration and stemness, which may shed new insight into HCC treatment.

## Background

There are more than 850,000 new cases of liver cancer every year worldwide [1]. Hepatocellular carcinoma (HCC) is the most common type of primary liver cancer and accounts for approximately 90% of liver cancer cases [2]. Hepatitis B virus (HBV) infection is the major risk factor for HCC, which lead to the high morbidity and mortality of HCC in China [3]. Despite great advancements for HCC therapies in recent years, the 5-year survival rate of HCC patients remains disappointing due to the metastasis and high recurrence rates [4]. Thus, it is in need to explore the molecular mechanisms underlying HCC and find novel therapeutic targets.

SEC61 translocon alpha 1 subunit (SEC61A1) was previously reported to contribute to progression of colon adenocarcinoma via the MNX1-AS1-miR-218-5p-SEC61A1 network [5]. The present study was designed to explore the function as well as the underlying mechanism of SEC61A1 in HCC.

Long non-coding RNAs (lncRNAs) are a group of non-coding RNAs with a length over 200 nucleotides, which have been reported to be involved in various biological processes of human cancers, including HCC. For examples, lncRNA ID2-AS1 suppresses HCC metastasis through activation of HDAC8/ID2 pathway [6]. Overexpression of lncRNA 91H accelerates HCC growth and metastasis via epigenetically activating IGF2 [7]. Macrophages-induced lncRNA H19 axis promotes HCC cell aggressiveness via miR-193b/MAPK1 axis [8]. PITPNA-AS1 blocks the suppression of miR-876-5p on WNT5A expression to facilitate HCC progression [9].

Competitive endogenous RNA (ceRNA) is a typical post-transcriptional mechanism in which lncRNAs positively regulated mRNAs by sponging miRNAs. LncRNAs have been widely reported as a ceRNA in tumorigenesis and tumor development. For instance, lncRNA LINC00899 inhibits progression of breast cancer by sequestering miR-425 to enhance DICER1 expression [10]. LncRNA DSCAM-AS1 strengthens cell proliferation, migration and invasion ability in cervical cancer through serving as a ceRNA to modulate miR-877-5p/ATXN7L3 axis [11]. LncRNA LINC00460 contributes to cell growth in head and neck squamous cell carcinoma through sponging miR-612 to up-regulate AKT2 [12]. LncRNA SPRY4-IT1 functions as a molecular sponge of miR-6882-3p to increase TCF7L2 expression in breast cancer [13]. Our current study was aimed at investigating the ceRNA network associating with the upregulation of SEC61A1 in HCC cells.

## Methods

### Cell culture

Human HCC cell lines (Hep3B, HCCLM3, Huh-7, MHCC97-L) and human normal liver epithelial cell line (THLE-3) purchased from ATCC (Manassas, VA, USA) were allowed to grow in DMEM culture medium (Invitrogen, Carlsbad, CA, USA) with 1% Pen/Strep mixture (Invitrogen) and 10% fetal bovine serum (FBS; Invitrogen). Cell culture was carried out in a humidified atmosphere of 5% CO_2_ and 37 °C.

### Quantitative real‐time PCR (qRT-PCR)

Total cellular RNAs were acquired with TRIzol reagent (Thermo Fisher Scientific, Waltham, MA, USA) as per the user guide. After denaturing for 5 min, RNAs were reversely transcribed into cDNA with TaKaRa Reverse Transcription Kit (Shiga, Japan). qPCR was conducted for gene expression analysis with Power SYBR Green (TaKaRa) in line with the manual. All results were processed with the comparative delta-delta CT method (2^−ΔΔCt^) with GAPDH or U6 as negative control.

### Transfection

The designed shRNAs and control-shRNAs were acquired from RiboBio (Guangzhou, China) to silence SEC61A1 and VPS9D1-AS1 using Lipofectamine2000 (Invitrogen). The miR-491-5p mimics and NC mimics, miR-491-5p-inhibitor and NC inhibitor, as well as pcDNA3.1-SEC61A1, pcDNA3.1-VPS9D1-AS1, pcDNA3.1-EGR1 and empty pcDNA3.1vector (negative control) were all designed by Genepharma Company (Shanghai, China). At 48 h post-transfection, HCCLM3 and Huh-7 cells were harvested.

### EdU assay

HCCLM3 and Huh-7 cells were transfected as study designed and put on sterile coverslips in the 24-well plates. EdU assay kit obtained from Ribobio was used for assessing cell proliferation as per instruction. Nuclei were detected via DAPI dye. Samples were imaged by a fluorescent microscope (Leica, Wetzlar, Germany).

### Colony formation assay

Transfected cells were placed in 6-well plates with 800 cells/well for 2 weeks. After fixing in 4% paraformaldehyde, cells were stained in 0.1% crystal violet, then imaged and counted.

### Transwell assay

Transwell assay was conducted following the previous study [14].

### Sphere formation assay

HCCLM3 and Huh-7 cells were seeded in 96-well ultralow attachment plates (Corning Inc., New York, NY) adding sphere medium. After 7-days of cell culture, images were taken, and sphere cells were counted.

### RNA pull down assay

RNA pull down assay were conducted using Pierce Magnetic RNA-Protein Pull-Down Kit (Thermo Fisher Scientific). Cell protein extracts were mixed with the biotin-labeled probes for SEC61A1 or miR-491-5p, then streptavidin agarose magnetic beads were added. After centrifugation, the obtained pull-downs were assayed by qRT-PCR.

### Luciferase reporter assay

The wild-type and mutated SEC61A1 or VPS9D1-AS1 fragments covering the miR-491-5p binding sites were separately inserted to pmirGLO luciferase vector. The obtained SEC61A1-WT/MUT and VPS9D1-AS1-WT/MUT were specifically co-transfected, then analyzed by luciferase reporter assay system (Promega, Madison, WI) after 48 h. For promoter analysis, HCC cells were co-transfected with pGL3-vector containing wild-type or mutated VPS9D1-AS1 promoter and pcDNA3.1-EGR1 or control.

### Subcellular fractionation

With PARIS™ Kit (Invitrogen), the separation of nuclear and cytoplasmic RNAs from HCC cells were achieved following the direction. For the subcellular location of VPS9D1-AS1, GAPDH and U6 were used as cytoplasmic and nuclear indicator, respectively.

### RNA immunoprecipitation (RIP)

RIP assay was performed in HCC cells using Magna RIP RNA-Binding Protein Immunoprecipitation Kit as instructed by supplier (Millipore, Bedford, MA). Anti-Ago2 antibody and control IgG antibody (Millipore) were employed to incubate RNA immunoprecipitate. RNA enrichment was assessed by qRT-PCR.

### Chromatin immunoprecipitation (ChIP)

After cross-linking in 4% formaldehyde for 10 min, samples were collected and re-suspended in the lysis buffer, sonicated, then incubated with anti-EGR1 or anti-IgG. After decrosslinking, the precipitates were subjected to qRT-PCR analysis.

### Statistical analyses

Each experiment with bio-triplicates was repeated at least three times. The results were displayed with mean ± standard deviation (SD). PRISM 6 (GraphPad, San Diego, CA) was utilized for data analysis. Differences between two groups were analyzed by Student’s t test, whereas those among multiple groups were analyzed by one-way ANOVA. Statistical significance was identified as p-values below 0.05.

## Results

### SEC61A1 promotes HCC cell proliferation, migration and stemness

**Fig. 1:**
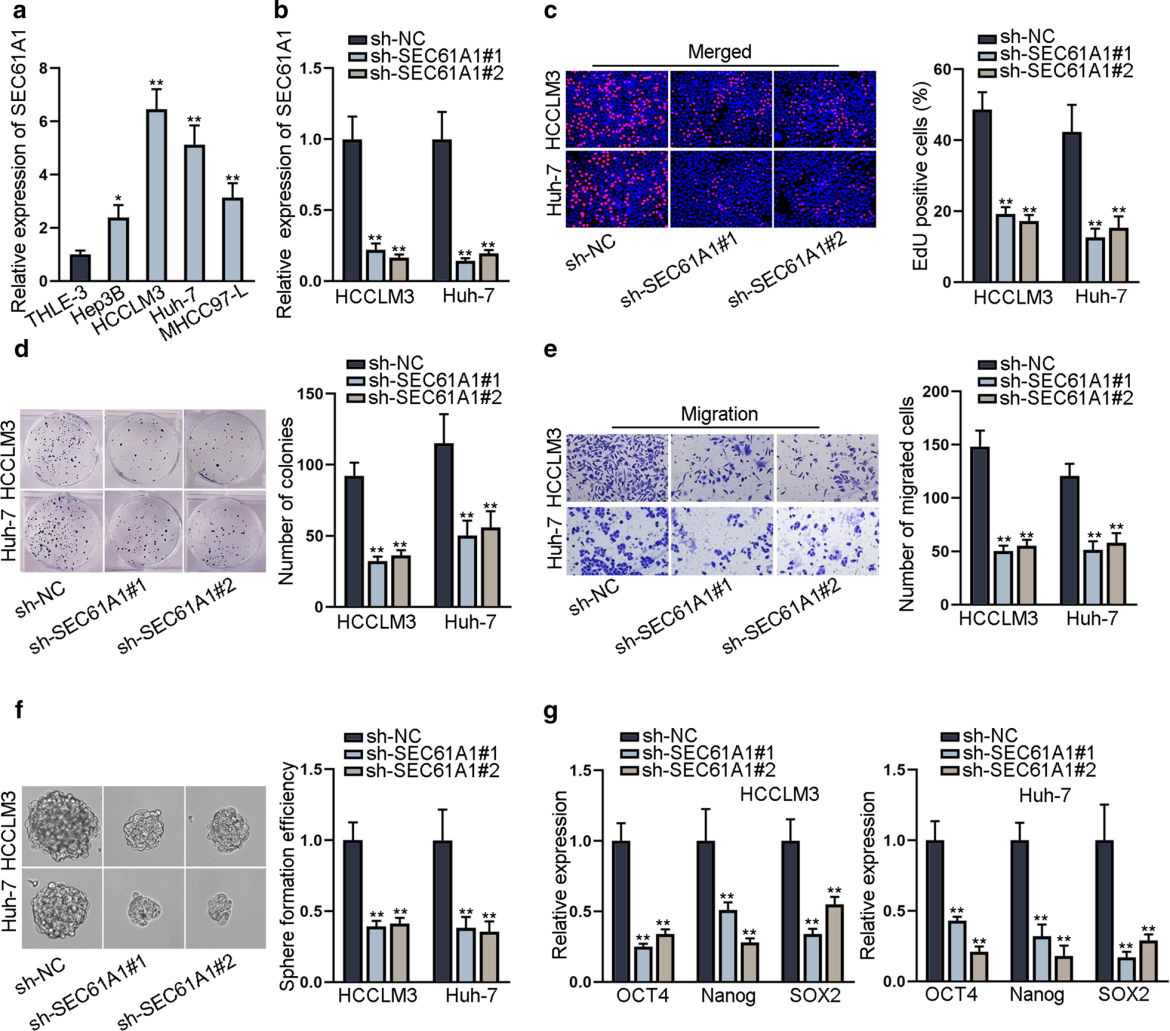
SEC61A1 promotes HCC cell proliferation, migration and stemness. **a** SEC61A1 expression in HCC cell lines and normal liver epithelial cell line was evaluated through qRT-PCR. **b** SEC61A1 expression was decreased in HCC cells transfected with sh-SEC61A1#1/2. **c**,** d** Proliferation ability of SEC61A1-silenced HCC cells was evaluated through EdU assay and colony formation assay. **e** Transwell assay examined the migration ability of HCC cells transfected with sh-SEC61A1#1/2 or sh-NC. **f** Sphere formation assay detected the sphere formation efficiency in SEC61A1-silenced HCC cells. **g** Stemness markers, including OCT4, Nanog and SOX2 were detected at the RNA level through qRT-PCR. *P < 0.05, **P < 0.01

To explore the role of SEC61A1 in HCC, the expression of SEC61A1 in HCC cell lines and normal liver epithelial cell line was evaluated. The results revealed that SEC61A1 was significantly up-regulated in HCC cell lines (Fig. 1a). For loss-of-function assays, SEC61A1 was silenced in HCCLM3 and Huh-7 cells with relative highest level of SEC61A1 (Fig. 1b). Based on the results of EdU and colony formation assays, we determined the depletion on cell proliferation after SEC61A1 knockdown (Fig. 1c, d). Moreover, the migratory ability of HCC cells was impaired by the knockdown of SEC61A1 (Fig. 1e). Sphere formation assay demonstrated that sphere formation efficiency was inhibited in SEC61A1 silenced HCC cells (Fig. 1f). Meanwhile, silencing of SEC61A1 led to the downregulation of stemness markers (OCT4, Nanog and SOX2) (Fig. 1g), further verified the inhibitory effect of SEC61A1 knockdown on stemness. Based on these data, we concluded that SEC61A1 promotes HCC cell proliferation, migration and stemness.

### SEC61A1 is a downstream target of miR-491-5p

**Fig. 2:**
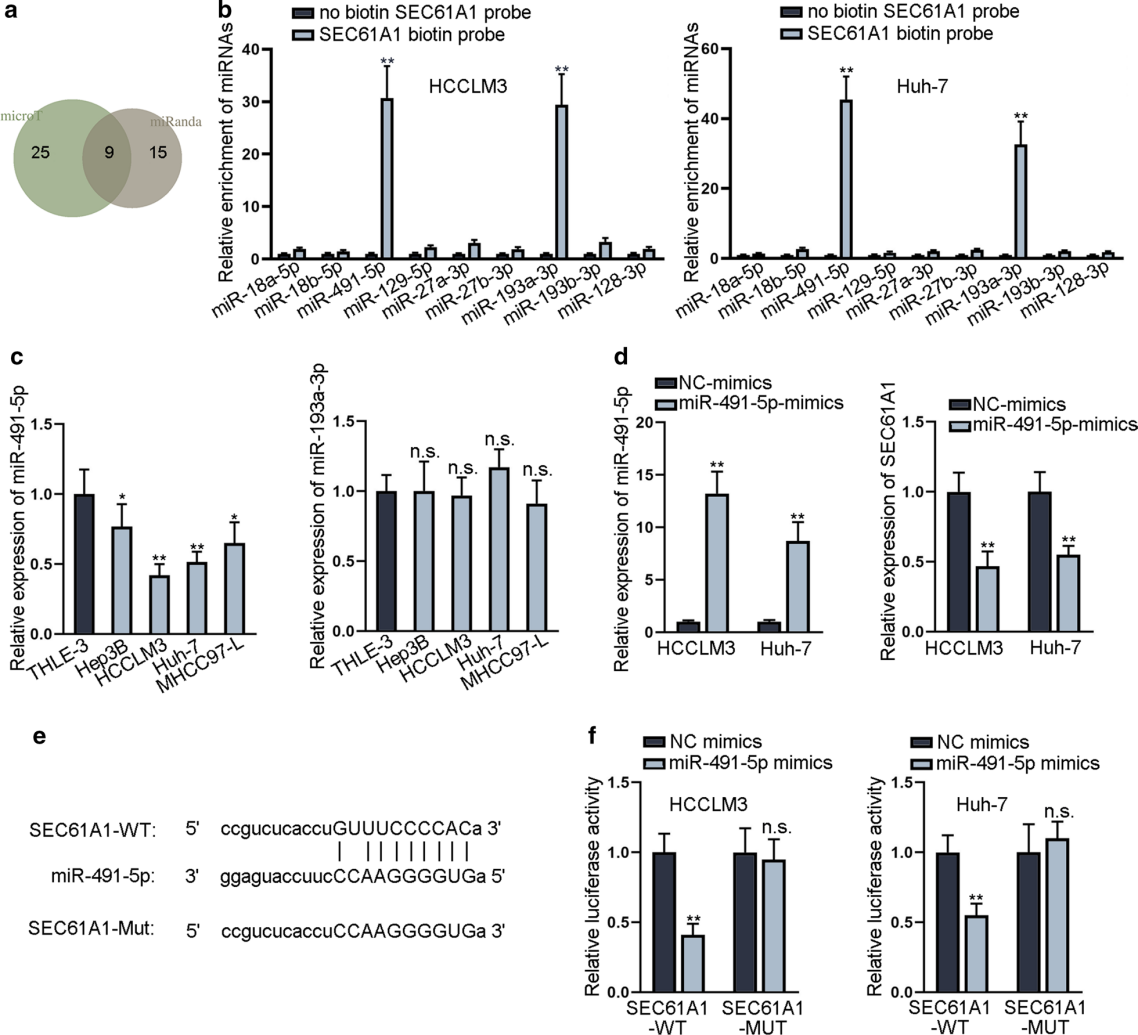
SEC61A1 is a downstream target of miR-491-5p. **a** The venn diagram revealed 9 miRNAs which targeted SEC61A1 based on prediction from 2 databases (microT and miRanda). **b** RNA pull down assay disclosed enrichment of 9 candidate miRNAs pulled down by biotin-labeled SEC61A1. **c** qRT-PCR examined expression of miR-491-5p and miR-193a-3p in HCC cells and normal liver epithelial cells. **d** qRT-PCR detected miR-491-5p and SEC61A1 expression under miR-491-5p overexpression. **e** “ENCORI” database predicted the binding sequences of SEC61A1 and miR-491-5p. **f** Luciferase reporter assay revealed the luciferase activity of wild and mutant SEC61A1 in response to miR-491-5p up-regulation. *P < 0.05, **P < 0.01. n.s. indicated difference was not statistically significant

Since mRNAs was usually regulated by their upstream miRNAs, we then explored the upstream miRNAs of SEC61A1 using ENCORI database [15]. Nine miRNAs were selected out based on 2 programs of microT and miRanda (Fig. 2a). Then, we conducted RNA pull down assay and proved that only miR-491-5p and miR-193a-3p were enriched in the products pulled down by biotin labeled SEC61A1 (Fig. 2b). Next, qRT-PCR analysis revealed that miR-491-5p was downregulated in HCC cells (Fig. 2c), whereas miR-193a-3p presented no significant expression difference in HCC cells and normal cell. Subsequently, we enhanced miR-491-5p expression in HCC cells and found that the expression of SEC61A1 was decreased (Fig. 2d). The binding sites of SEC61A1 and miR-491-5p were predicted and mutated for subsequent analysis (Fig. 2e). Luciferase reporter assay disclosed that luciferase activity of wild SEC61A1 was decreased by miR-491-5p-mimics (Fig. 2f). Thus, we confirmed that SEC61A1 is a downstream target of miR-491-5p in HCC cells.

### VPS9D1-AS1 serves as a sponge of miR-491-5p to up-regulate SEC61A1

**Fig. 3:**
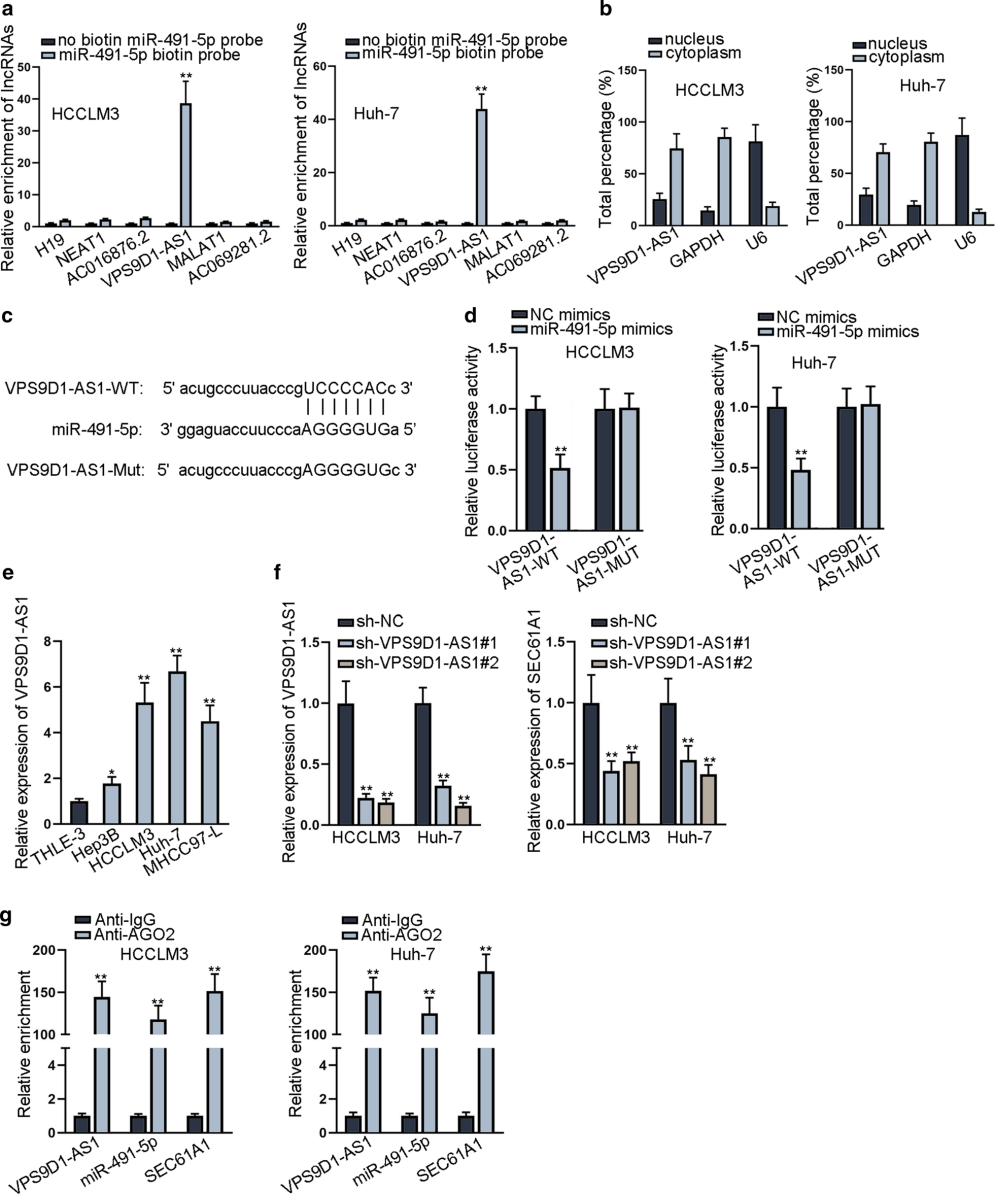
VPS9D1-AS1 serves as a sponge of miR-491-5p to up-regulate SEC61A1. **a** RNA pull down assay demonstrated enrichment of 6 candidate lncRNAs pulled down by biotin-labeled miR-491-5p. **b** Nucleus/cytoplasm fraction assay revealed subcellular location of VPS9D1-AS1. **c** Binding sites between VPS9D1-AS1 and miR-491-5p were predicted from “ENCORI” database. **d** Luciferase reporter assay revealed the luciferase activity of wild and mutant VPS9D1-AS1 in cells co-transfected with miR-491-5p mimics or NC mimics. **e** Relative expression of VPS9D1-AS1 in HCC cells and normal liver epithelial cells was evaluated by qRT-PCR. **f** Depletion efficiency of VPS9D1-AS1 in HCC cells and relative SEC61A1 expression were revealed in qRT-PCR. **g** RIP assay detected enrichment of VPS9D1-AS1, miR-491-5p and SEC61A1 pulled down by anti-AGO2 and anti-IgG. *P < 0.05, **P < 0.01

Since lncRNAs are reported as miRNAs’ sponges to upregulate mRNAs, we explored whether the inhibitory effect of miR-491-5p on SEC61A1 could be attenuated by a certain lncRNA. Seven candidate lncRNAs were screened out from ENCORI database with strict stringency of CLIP data and 4 cancer types in Pan-cancer. As revealed in RNA pull down assay, only VPS9D1-AS1 was significantly pulled down by biotin-labeled miR-491-5p (Fig. 3a). Importantly, VPS9D1-AS1 was mainly distributed in the cytoplasm of HCC cells (Fig. 3b). The binding sites of VPS9D1-AS1 and miR-491-5p were then predicted and mutated for the following assays (Fig. 3c). The luciferase activity of wild type VPS9D1-AS1 was decreased by miR-491-5p mimics (Fig. 3d), suggesting the interaction between VPS9D1-AS1 and miR-491-5p. Then, we verified the significant up-regulation of VPS9D1-AS1 in HCC cells (Fig. 3e). Additionally, we determined that silencing of VPS9D1-AS1 led to the downregulation of SEC61A1 in HCC cells (Fig. 3f). Further, RIP assay disclosed that VPS9D1-AS1, miR-491-5p and SEC61A1 were abundantly enriched in anti-AGO2 group, suggesting that VPS9D1-AS1, miR-491-5p and SEC61A1 were adhered to RISC (Fig. 3i). Taken together, VPS9D1-AS1 serves as a ceRNA to up-regulate SEC61A1 via sponging miR-491-5p.

### VPS9D1-AS1 promotes HCC cell proliferation, migration and stemness via regulating miR-491-5p and SEC61A1

**Fig. 4:**
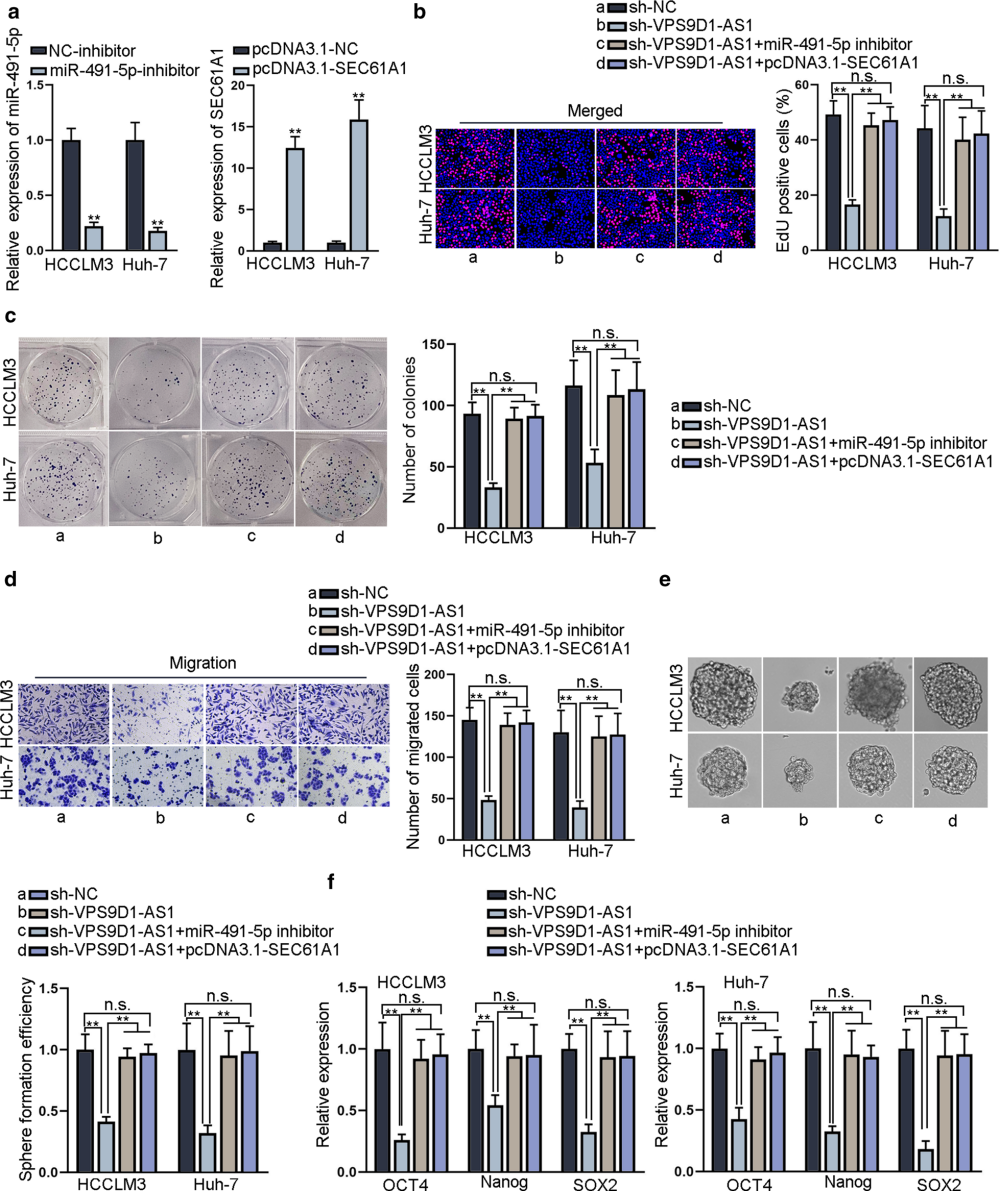
VPS9D1-AS1 promotes HCC cell proliferation, migration and stemness via regulating miR-491-5p and SEC61A1. **a** qRT-PCR verified the efficiency of miR-491-5p depletion and SEC61A1 overexpression in HCC cells. **b**, ** c** EdU and colony formation assay detected HCC cell proliferation ability in sh-NC, sh-SEC61A1, sh-VPS9D1-AS1 + miR-491-5p inhibitor and sh-VPS9D1-AS1 + pcDNA3.1-SEC61A1 groups. **d** Transwell assay detected migration ability of HCC cells in different four groups. **e**, ** f** Sphere formation and qRT-PCR assay evaluated HCC cell stemness in different four groups. **P < 0.01

To confirm the function of VPS9D1-AS1/miR-491-5p/SEC61A1 ceRNA network, rescue assays were carried out. We firstly silenced miR-491-5p and overexpressed SEC61A1, respectively (Fig. 4a). Then, EdU assay and colony formation assay revealed that HCC cell proliferation ability was hindered by VPS9D1-AS1 depletion, but such effects were reversed by the inhibition of miR-491-5p or overexpression of SEC61A1 (Fig. 4b, c). Next, transwell assay disclosed that down-regulation of miR-491-5p or up-regulation of SEC61A1 restored VPS9D1-AS1 depletion-mediated suppression on HCC cell migration (Fig. 4d). Further, sphere formation assay and qRT-PCR detection of stemness biomarkers demonstrated that HCC cell stemness hindered by VPS9D1-AS1 silence was recovered by miR-491-5p down-regulation or SEC61A1 up-regulation (Fig. 4e, f). Based on these findings, we draw the conclusion that VPS9D1-AS1 promotes HCC cell proliferation, migration and stemness via miR-491-5p/SEC61A1 axis.

### EGR1 transcriptionally activates SEC61A1 and VPS9D1-AS1

**Fig. 5:**
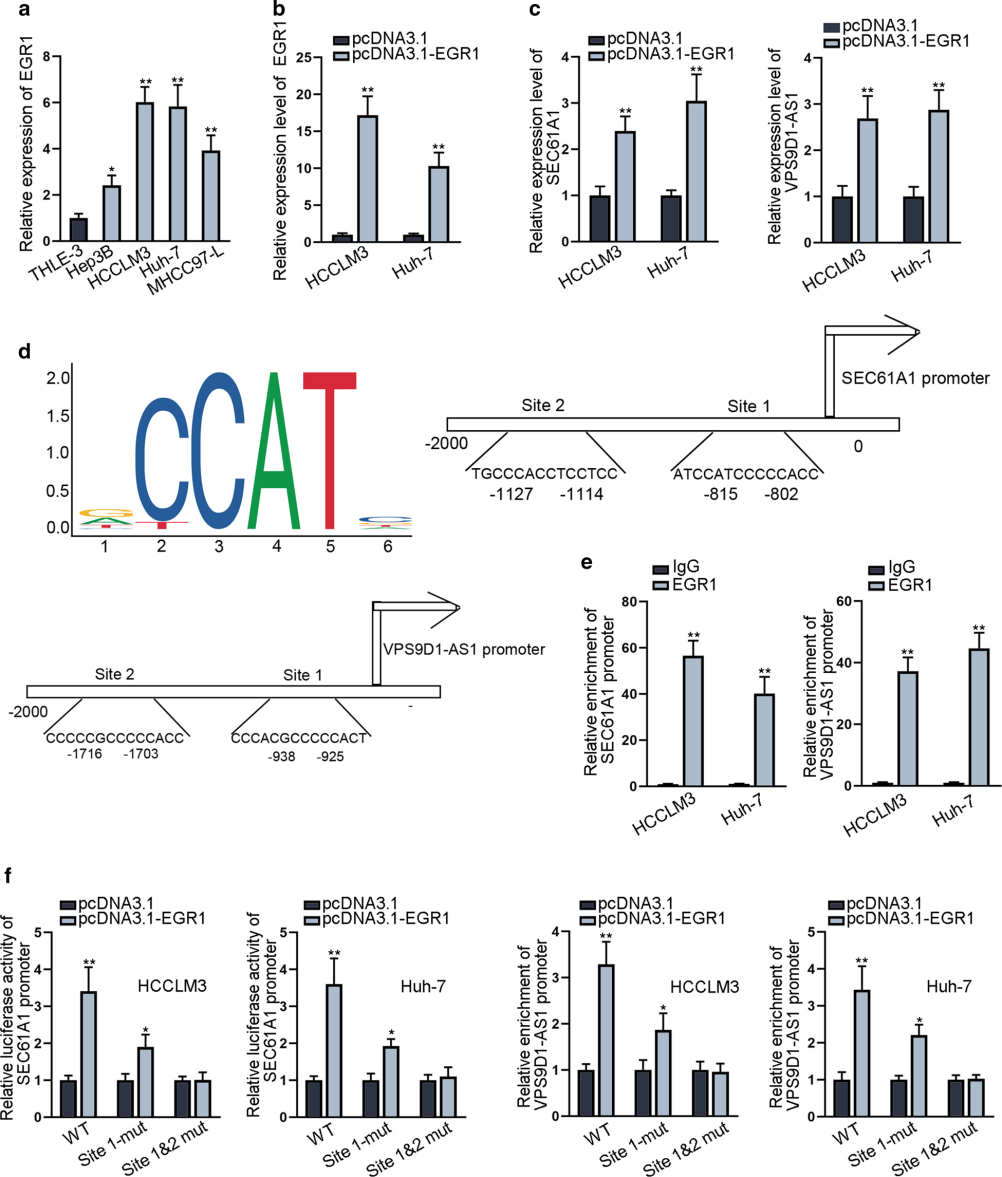
EGR1 transcriptionally activates SEC61A1 and VPS9D1-AS1. **a** qRT-PCR evaluated relative expression of VPS9D1-AS1 in HCC cells and normal liver epithelial cells. **b** Overexpression efficiency of EGR1 was verified in qRT-PCR. **c** Influence of EGR1 up-regulation on SEC61A1 and VPS9D1-AS1 expression was explored by qRT-PCR assay. **d** DNA motif of EGR1 and the binding sites of EGR1 on promoter of SEC61A1 and VPS9D1-AS1 were exhibited. **e** ChIP assay verified relative enrichment of SEC61A1 and VPS9D1-AS1 promoter pulled down by anti-EGR1. **f** Luciferase reporter assay revealed luciferase activity of wild and mutant SEC61A1 promoter and VPS9D1-AS1 promoter by treatment of pcDNA3.1-EGR1. *P < 0.05, **P < 0.01

Considering the upregulation of VPS9D1-AS1 and SEC61A1 in HCC cells, we also investigated the potential upstream molecular mechanism. At first, we identified the upregulation of EGR1 in HCC cells (Fig. 5d). Then, we overexpressed EGR1 (Fig. 5b) and observed the upregulation of VPS9D1-AS1 and SEC61A1 **(**Fig. 5c**)**. Based on UCSC and JASPAR databases, EGR1 was identified as the transcription factor for both VPS9D1-AS1 and SEC61A1. The DNA motif and putative binding sites of EGR1 on promoters of VPS9D1-AS1 and SEC61A1 were illustrated in Fig. 5d. Moreover, ChIP assay illustrated the abundant enrichment of VPS9D1-AS1 and SEC61A1 promoter in the immunoprecipitation pulled down by anti-EGR1 (Fig. 5e). Further, the luciferase activity of wild type VPS9D1-AS1 promoter and SEC61A1 promoter was increased by EGR1 up-regulation, whereas this tendency was abolished when the binding site 1 and 2 were both mutated (Fig. 5f), suggesting these two binding sites were responsible for the binding of EGR1 to SEC61A1 and VPS9D1-AS1 promoters. According to these data, we concluded that SEC61A1 and VPS9D1-AS1 are transcriptionally activated by EGR1.Luciferase reporter assay revealed luciferase activity of wild and mutant SEC61A1 promoter and VPS9D1-AS1 promoter by treatment of pcDNA3.1-EGR1. *P < 0.05, **P < 0.01.

## Discussion

HCC is a prevalent cancer in China and has severely threatens the public health. Despite the improvements on HCC treatment, the prognosis of patients remains unfavorable. It is quite important to reveal the molecular mechanism underlying the progression of HCC and thus find novel therapeutic targets. This study detected the function of SEC61A1 in HCC and explored its underlying molecular mechanism. As is revealed by Li N et al., SEC61A1 may be a prognostic marker for patients with HCC [16]. However, the specific role of SEC61A1 in HCC remains covered. In the current study, we figured out that SEC61A1 was up-regulated in HCC cells and promoted HCC cell proliferation, migration and stemness.

Then, we identified miR-491-5p as the up-stream suppressor of SEC61A1. MiR-491-5p negatively regulated SEC61A1 expression by directly targeting to its 3’UTR. Xu Q et al. have revealed that decreased expression of miR-491-5p is correlated with unfavorable prognosis of HCC patients [17]. MiR-491-5p functions as the tumor suppressor in colorectal cancer [18] and osteosarcoma [19]. Similarly, in this study, we determined that miR-491-5p exerted tumor-suppressive functions in HCC through targeting SEC61A1.

The ceRNA pattern has been widely reported in HCC. For examples, lncRNA RHPN1-AS1 drives HCC progression via blocking suppression of miR-596 on IGF2BP2 expression [20]. LncRNA ANRIL enhances mitochondrial function of HCC via mediation on miR-199a-5p/ARL2 axis [21]. LncRNA SSTR5‑AS1 contributes to development of HBV‑related HCC serving as a ceRNA to regulate CA2 through sponging miR‑15b‑5p [22]. Overexpression of PIK3CD-AS1 inhibits cell growth, invasion and migration of HCC by competitively binding microRNA-566 to promote expression of LATS1 [23]. Here, lncRNA VPS9D1-AS1 was verified as the sponge of miR-491-5p. VPS9D1-AS1 elevated SEC61A1 expression via sponging miR-491-5p but not regulation on miR-491-5p. Up-regulation of VPS9D1-AS1 is reported to predict poor prognosis in non-small cell lung cancer [24]. VPS9D1-AS1 serves as the ceRNA to promote cell proliferation in prostate cancer via sponging miR-184 to up-regulate c-Myc [25]. Present study uncovered that VPS9D1-AS1 promoted HCC cell proliferation, migration and stemness via miR-491-5p down-regulation and SEC61A1 up-regulation. EGR1 has been reported to induce upregulation of lncRNA FOXD2-AS1, which promotes the progression of HCC via activation of Wnt/β-catenin pathway [26]. In this study, EGR1 was identified to transcriptionally activate both VPS9D1-AS1 and SEC61A1. In conclusion, our current study revealed a novel ceRNA pathway in HCC, which may contribute to finding novel therapeutic targets for HCC patients. Lack of investigation on human subjects is a shortcoming of our current study, we will analyze clinical significance of EGR1/VPS9D1-AS1/miR-471-5p/SEC61A1 pathway in our future study.

## Conclusions

Present study uncovered a novel ceRNA network constituted by VPS9D1-AS1, miR-491-5p and SEC61A1 in HCC. Moreover, VPS9D1-AS1 and SEC61A1 were transcriptionally activated by EGR1. VPS9D1-AS1 contributes to HCC cell proliferation, migration and stemness via SEC61A1 up-regulation in a miR-491-5p-dependent way, indicating VPS9D1-AS1 as the putative therapeutic target for HCC treatment.

## Data Availability

Not applicable.

## Abbreviations

HCC: Hepatocellular carcinoma
lncRNA: Long non-coding RNA
VPS9D1-AS1: VPS9D1 antisense RNA 1
ceRNA: Competing endogenous RNA
miRNA: MicroRNA
EGR1: Early growth response 1
SEC61A1: SEC61 translocon alpha 1 subunit
qRT-PCR: Quantitative real-time PCR
RIP: RNA binding protein immunoprecipitation
ChIP: Chromatin immunoprecipitation
shRNA: Short hairpin RNA
FBS: Fetal bovine serum
ATCC: American Type Culture Collection
RISC: RNA induced silence complex
